# *Propionibacterium acnes* induces intervertebral disc degeneration by promoting nucleus pulposus cell apoptosis via the TLR2/JNK/mitochondrial-mediated pathway

**DOI:** 10.1038/s41426-017-0002-0

**Published:** 2018-01-10

**Authors:** Yazhou Lin, Yucheng Jiao, Ye Yuan, Zezhu Zhou, Yuehuan Zheng, Jiaqi Xiao, Changwei Li, Zhe Chen, Peng Cao

**Affiliations:** 1 Department of Orthopedics, Ruijin Hospital, Shanghai Jiaotong University School of Medicine, Shanghai, 200000 China; 2 Shanghai Key Laboratory for Prevention and Treatment of Bone and Joint Diseases with Integrated Chinese-Western Medicine, Shanghai Institute of Traumatology and Orthopedics, Ruijin Hospital, Shanghai Jiaotong University School of Medicine, Shanghai, 200000 China; 3 Department of Orthopedics, Xinhua Hospital, Shanghai Jiaotong University School of Medicine, Shanghai, 200000 China; 4 Department of Orthopedics, Ruijin Hospital North, Shanghai Jiaotong University School of Medicine, Shanghai, 200000 China; 5 Department of Medical Microbiology and Parasitology, Shanghai Jiaotong University School of Medicine, Shanghai, 200000 China

## Abstract

Evidence suggests that intervertebral disc degeneration (IVDD) can be induced by *Propionibacterium acnes* (*P. acnes*), although the underlying mechanisms are unclear. In this study, we analyzed the pathological changes in degenerated human intervertebral discs (IVDs) infected with *P. acnes*. Compared with *P. acnes*-negative samples, *P. acnes*-positive IVDs showed increased apoptosis of nucleus pulposus cells (NPCs) concomitant with severe IVDD. Then, a *P. acnes*-inoculated IVD animal model was established, and severe IVDD was induced by *P. acnes* infection by promoting NPC apoptosis. The results suggested that *P.acnes*-induced apoptosis of NPCs via the Toll-like receptor 2 (TLR2)/c-Jun N-terminal kinase (JNK) pathway and mitochondrial-mediated cell death. In addition, *P. acnes* was found to activate autophagy, which likely plays a role in apoptosis of NPCs. Overall, these findings further validated the involvement of *P. acnes* in the pathology of IVDD and provided evidence that *P. acnes*-induced apoptosis of NPCs via the TLR2/JNK pathway is likely responsible for the pathology of IVDD.

## Introduction

Intervertebral disc degeneration (IVDD) produces a series of clinical symptoms, such as sciatica, low back pain and physical dysfunction, all of which drastically affect quality of life and work productivity of affected individuals and significantly increase the burden of medical treatment^1^. However, the etiology and pathophysiological mechanisms of IVDD are not well understood and emphasize the need to further investigate IVDD for better therapy.

Traditionally, excessive mechanical loading, a nutritional disorder, traumatic injury or genetic predisposition is considered the main etiology for IVDD^1^. Recent studies have proposed “bacteria-induced disc degeneration”^2^ because low-virulence anaerobic bacteria, such as *P. acnes*, were found to latently reside inside non-pyogenic IVD. In these patients, the prevalence of *P. acnes* in IVDD ranged from 13 to 44%^3–7^. Further epidemiologic investigation and animal experiments suggested a link between bacterial infection and IVDD^8–11^. Therefore, the theory of bacterial disc degeneration has drawn increasing attention.

*P. acnes*, a microaerophilic or anaerobic gram-positive rod-shaped bacterium, is an important opportunistic pathogen that causes several diseases, such as endocarditis, prostate cancer, prosthetic joints, and orthopedic device-related infections and sarcoidosis^12^. Moreover, a growing body of evidence suggests that *P. acnes* is capable of growing and reproducing inside IVD^11^. In our previous study, *P. acnes* colonies were identified in non-pyogenic degenerated IVD by anaerobic culture and histological observation, and the prevalence rate of *P. acnes* in IVDD was 21.05% (16/76)^13^. Additionally, inoculation of *P. acnes* into normal rabbit IVDs induced severe disc degeneration and Modic changes^8^. Therefore, *P. acnes* was thought to be a potential pathogenic factor for IVDD. However, the mechanisms by which *P. acnes* induce IVDD are unclear.

Studies have suggested that cellular loss caused by excessive apoptosis of disc cells, especially the death of nucleus pulposus cells (NPCs), could play an important role in IVDD^14–16^. NPCs are known to resist mechanical loading by synthesizing extracellular matrix (ECM) and thus maintaining the stability of IVD. Many complex and interdependent factors have been implicated in the excessive apoptosis of NPCs^15^. Thus far, studies examining the apoptotic signal transduction pathways of IVD cells have mainly focused on three apoptosis signaling pathways: the mitochondrial pathway, death receptor pathway and endoplasmic reticulum (ER) pathway^15^. The mitochondrial pathway is activated by various cellular stresses and numerous apoptotic signals and is important for IVD cell apoptosis, which occurs during IVD degeneration^17^.

Because the etiology of *P. acnes*-induced IVDD is less well understood and the death of NPCs plays an important role in IVDD, here we investigated the potential relationship between *P. acnes* infection and NPC apoptosis. We also explored the specific signaling pathway responsible for the apoptosis of NPCs. To our knowledge, this is the first study to investigate the relationship between *P. acnes* infection and NPC apoptosis, and our findings provide new insights for the prevention and treatment of degenerative disc diseases.

## Materials and methods

### Patients and tissue harvesting

A total of 108 patients were included in this study conducted from September 2013 to May 2017. The patients underwent discectomy at the single-level lumbar spine due to disc degeneration associated with low back pain and/or sciatica. All patients had decided on surgery after failed attempts to improve their condition using conservative treatment for several months. Patients who received antibiotics within the month preceding surgery were not included in this study. The average age of patients included in the study was 56.78 ± 14.59 years, and 60 patients were male and 48 patients were female. The levels of surgery were as follows: 3 at L2~3, 12 at L3~4, 63 at L4~5, and 30 at L5~S1. The study was approved by the Institutional Review Board of Shanghai Ruijin Hospital and informed consent forms were signed by all patients.

Based on a stringent antiseptic sterile protocol described in our previous study, a posterior discectomy was performed to harvest IVD^9,13^. Briefly, the skin of the operation field was sterilized three times with povidone iodine, and a 3 M Ioban 2 Antimicrobial Incise Drape (3 M Health Care, St. Paul, MN, USA) was used to cover the surgical field. The wound was then irrigated twice using sterile water before discectomy of the IVD. The harvested specimen was handled exclusively with sterilized instruments to avoid contamination. Finally, some muscle and ligament samples adjacent to the IVD were collected after discectomy to serve as markers of contamination and were cultured under the same conditions as the harvested IVDs.

### Bacterial culture and 16Sr PCR

First, all tissues were cultured in tryptone soy broth for 14 days under anaerobic conditions (80% N_2_, 10% CO_2_, 10% H_2_, 37 °C). Then, the presence of bacteria in the culture was identified by amplifying the 16S rDNA gene by PCR according to our previous protocol^13^. Specific primers targeting *P. acnes* were designed. Forward primer: 5′-GGG TTG TAA ACC GCT TTC GCC T-3′ Reverse primer: 5′-GGC ACA CCC ATC TCT GAG CAC-3′.

### **Preparation of*P. acnes*inoculum**

A standard strain of *P. acnes* (ATCC: 6919, GIM: 1.243, Guangdong Microbiology Culture Center, Guangdong, China) was cultured on Gifu Anaerobic (GAM) broth (Nissui, Tokyo, Japan) for 3 d at 37 °C under anaerobic conditions.

### Inoculation of *P. acnes* into caudal rat intervertebral discs

Eight-week-old male Sprague-Dawley rats were purchased from the Shanghai Laboratorial Animal Center at the Chinese Academy of Sciences. The animals were housed with ad libitum access to water and food in an air-conditioned room with a 12-h light–dark cycle, at 21 to 23 °C and 60% relative humidity, in the animal facility at Ruijin Hospital, Shanghai Jiao Tong University School of Medicine, China. Rats were anesthetized intraperitoneally with 2.5% sodium pentobarbital (1.3 mL/kg) and placed in a prone position, with 4 rats per group. Then, the tail skin was sterilized with 75% alcohol three times. Before surgery, the target vertebrae (Ca) 6/7 to (Ca) 8/9 (*n* = 3 per animal) were identified and marked by palpation and X-ray. The diameters of the target IVD were measured using X-ray before surgery to determine the depth of puncture. A volume of 2.5 µL *P. acnes* (OD_600_ = 3.0), *P. acnes* with Z-VAD-FMK (caspase protein inhibitor, 1.5 mM, NO C1202, Beyotime, Shanghai, China) or saline was inoculated vertically into the nucleus pulposus using a microsyringe with a 28-gauge needle (Hamilton, Nevada, USA). The penetration depth was fixed at 2.0–2.5 mm using a stopper. All animal experiments were performed in accordance with the protocol approved by the Shanghai Jiao Tong University (SJTU) Animal Care and Use Committee [IACUC protocol number: SYXK (Shanghai)2011–0113] and in accordance with the Ministry of Science and Technology of the People’s Republic of China Animal Care guidelines. All surgeries were performed under anesthesia, and all efforts were made to minimize suffering.

### **Co-cultures of NPCs and*P. acnes***

Nucleus pulposus tissues were harvested and cultured from six disc degenerated patients, including four males and two females, with a mean age of 36.5 years (28–50 years) following the above protocol. Cell samples from different patients were kept separate. All experiments were carried out in duplicate and were conducted with human NPCs from passages two to three.

For co-culture, the bacteria were harvested from 3-d cultures in stationary phase and washed twice with phosphate-buffered saline (PBS). The bacterial density was adjusted to optical density (OD = 2). Then, *P. acnes* were added to the cell culture (5 × 10^5^ cells/well) in a 6-well culture plate at a 100:1 multiplicity of infection (MOI) without antibiotics. After 1, 4, 8, 16, and 24 h, co-cultured cells were washed three times with PBS and prepared for late-stage experiments.

### Western blot analysis

For western blot analysis, total proteins from the samples were separated by SDS-PAGE, transferred to nylon membranes and incubated separately with the following primary antibodies: Collagen II (dilution of 1: 2000; cat. NO ab34712, Abcam, Britain), Aggrecan (dilution of 1: 1000; cat. NO ab36861, Abcam, Britain), LC-3A/B (dilution of 1: 1000; cat. NO 12741S, CST, Inc., MA, USA), P62 (dilution of 1: 1000; cat. NO 8025S, CST, Inc., MA, USA), Beclin-1 (dilution of 1: 1000; cat. NO 3495S, CST, Inc., MA, USA), Bcl-2 (dilution of 1: 1000; cat. NO 3498S, CST, Inc., MA, USA), Bax (dilution of 1: 1000; cat. NO 5023S, CST, Inc., MA, USA), cleaved caspase-3 /-8 (dilution of 1: 1000; cat. NO 9664S/ 9496S, CST, Inc., MA, USA), Fas/FasL (dilution of 1: 1000; cat. NO 4233S/4273S, CST, Inc., MA, USA), mTOR/Phospho-mTOR (dilution of 1: 1000; cat. NO 2983S/5536S, CST, Inc., MA, USA), PI3 Kinase Class III (dilution of 1: 1000; cat. NO 4263S, CST, Inc., MA, USA), NF-κΒ/ Phospho-NF-κΒ (dilution of 1: 1000; cat. NO 8242S/3033S, CST, Inc., MA, USA), and MAPK family kit/P-MAPK family kit (dilution of 1: 1000; cat. NO 9926T/9910T, CST, Inc., MA, USA). B-actin (dilution of 1: 2000; cat. NO CW0096, CW BIO, Beijing, China) was used as an internal control. Then, the membranes were incubated with horseradish peroxidase-conjugated secondary antibody, goat anti-rabbit IgG (dilution, 1: 2000; cat. NO CW0103s; CW Bio, Beijing, China) or goat anti-mouse IgG (dilution, 1: 2000; cat. NO CW0102s; CW Bio, Beijing, China) at room temperature for 2 h, and the bands were visualized using chemiluminescence (Pierce Biotechnology, Inc., IL, USA). The images were analyzed using Fusion FX7 (Vilber Lourmat, Marne-la-Vallée, France).

### Quantification of gray value and intervertebral height

According to a previous study, variables of age, primary symptoms, duration of symptoms and surgery level dramatically affect the severity of IVDD. Thus, to reveal the true effects of *P. acnes* and reduce heterogeneity, a case-controlled method was used for the quantitative analysis following a previous study^18^. Briefly, after culture of the specimen and bacterial identification, the patients who had *P. acnes* only in IVD were classified as the positive group. Equal numbers of patients who were identified as completely bacteria-free in their IVD were selected to match each of the positive patients based on the following criteria: (1) same gender; (2) same surgery segment; (3) same symptoms of low back pain only, sciatica only or both; (4) similar ages ±5 years; (5) similar duration of symptoms ±3 months. These patients were named the negative group, and their demographics are listed in Supplementary Table S1.

The intervertebral height was measured by preoperative lateral X-ray following the distortion-compensated roentgen analysis method^19^. Briefly, a midplane connecting the ventral and dorsal midpoints of the vertebra was established, and the sagittal plane angle between two adjacent vertebrae was determined by the angle between their midplanes. Then, the ventral height of a lumbar disc was measured and corrected according to the sagittal plane angle. The resultant angle-standardized disc height was independent of the patient posture and more accurate. All evaluations were conducted independently by two examiners who were blinded to the groups.

To measure the gray value in IVD, the largest closed area between two adjacent vertebrae represented the NP and was selected as the region of interest. The MRI index was calculated as the sum of the pixel area multiplied by the pixel intensity for all identified NP tissues.

### Histological examination

IVD harvested from patients or rats were fixed in 4% formaldehyde for 24 h, processed by routine paraffin-embedding and sectioned at 5 μm. H&E staining, Safranin-O/fast green staining and Picrosirius Red staining was performed following the manufacturer’s instructions (Leagene Biotech CO. Ltd., Beijing, China). To detect the presence of bacteria, the stained samples were observed under a microscope with an oil immersion lens at a magnification of ×630 (Axio, Carl Zeiss, Oberkochen, Germany). Samples stained with Picrosirius Red were observed under a polarization microscope at a magnification of ×100 (Axio, Carl Zeiss, Oberkochen, Germany).

### Immunofluorescence

The disc samples harvested from patients or rats were embedded in Tissue-Tek (Sakura, CA, USA) and then sectioned at a thickness of 5 μm in the coronal plane using a freezing microtome (Leica CM1950, Leica Biosystems, Wetzlar, Germany). To prepare NPCs, *P. acnes*-induced cells were cultured on glass slides and then fixed for 30 min in 4% para-formaldehyde.

For apoptosis, the tissue sections or cells were stained for terminal deoxynucleotidyl transferase dUTP nick end labeling (TUNEL, Nanjing KeyGEN Biotech Co. Ltd., Nanjing, China) according to the manufacturer’s instructions to detect positive cells and determine microscopic counts. For autophagy, the NPCs were incubated for 16 h at 4 °C with LC3 antibody (1:100; cat. NO 12741S, CST, Inc., MA, USA). All images were observed using a fluorescence microscope (Axio, Carl Zeiss, Oberkochen, Germany).

### Flow cytometry, Caspase-3/-9 activity analysis and mitochondrial membrane potential measurements

Caspase-3/-9 activity was measured using the Caspase-3/-9 Colorimetric Assay Kit (BioVision, Inc., CA, USA) according to the manufacturer’s instructions. The OD of the samples were read at 405 nm using a microtiter plate reader (Sunrise™; Tecan Group, Ltd., Männedorf, Switzerland).

The proportion of NPC apoptosis was detected using the Annexin V-FITC apoptosis detection kit and calculated by the percentage of early apoptotic (Annexin V+/PI−) cells plus the percentage of late apoptotic (Annexin V+/PI+) cells using flow cytometry.

The mitochondrial membrane potential was determined by JC-1 staining, which is a dual-emission potential-sensitive probe, according to the manufacturer’s instructions. The cells were observed using an OLYMPUS BX51 microscope (OLYMPUS, Tokyo, Japan).

### Transfection of siRNA-Toll-like receptor 2 (TLR2) and siRNA-*Beclin1*

Cells were transfected with siRNAs-*Tlr2*, siRNA-*Beclin1* or with control siRNA using Lipofectamine® 3000 (Lipo3000, Thermo Fisher Scientific, Inc., MA, USA) at 37 °C in a humidified incubator with 5% CO_2_. The siRNAs were designed and synthesized by Shanghai GenePharma Co., Ltd. (Shanghai, China). Another group of cells was transfected with a GFP-labeled nonspecific siRNA that served as the negative control (NC). The sequences of the siRNAs used in the present study were as follows: siRNA-*Tlr2* sense: 5′-CAG AUC UAC AGA GCU AUG ATT-3′, anti-sense: 5′-UCA UAG CUC UGU AGA UCU GTT-3′; siRNA-*Beclin1* sense: 5′-GUG GAA UGG AAU GAG AUU ATT-3′, anti-sense: 5′-UAA UCU CAU UCC AUU CCA CTT-3′; NC-siRNA sense: 5′-UUC UCC GAA CGU GUC ACG UTT-3′, anti-sense: 5′-ACG UGA CAC GUU CG GAG AAT T -3′. When NPCs seeded into 6-well plates reached 80% confluence, transfection was performed by mixing 5 µL siRNA with 5 µL Lipo3000 in a final volume of 2000 µL DMED/F12 medium (Gibco; Thermo Fisher Scientific, Inc.) containing 15% serum without antibiotics, according to the manufacturer’s protocol. After 16 h of transfection, the cells were infected with *P. acnes* for 8 h. Finally, the mRNA and protein were extracted from the cells. Transfections were performed in triplicate, and the experiment was repeated at least three times.

### **Electron microscopy**

For electron microscopy, cells infected with *P. acnes* on chamber slides were fixed with 2.5% glutaraldehyde in 0.1 M phosphate buffer (pH 7.4) for 2 h. Conventional electron microscopy was performed as follows. After five washes with 0.1 M phosphate buffer, the cells were postfixed with 2% osmium tetroxide and 0.5% potassium ferrocyanide in the same buffer for 1 h and then washed again with 0.1 M phosphate buffer. After dehydration, the cells were embedded in Epon 812 (TAAB Laboratories Equipment Ltd.). Ultrathin sections were stained with uranyl acetate plus lead citrate and observed using an H7700 electron microscope (Hitachi, Tokyo, Japan).

### Statistical analysis

Data were collected from three or more independent experiments and expressed as the mean ± S.D. A two-sided Student’s *t* test was used to analyze differences between two groups. One-way analysis of variance was performed to show differences among multiple groups. *P* < 0.05 was considered significantly different.

## Results

### IVDs infected with *P. acnes* had more apoptotic NPCs concomitant with severe disc degeneration in patients

**Fig. 1:**
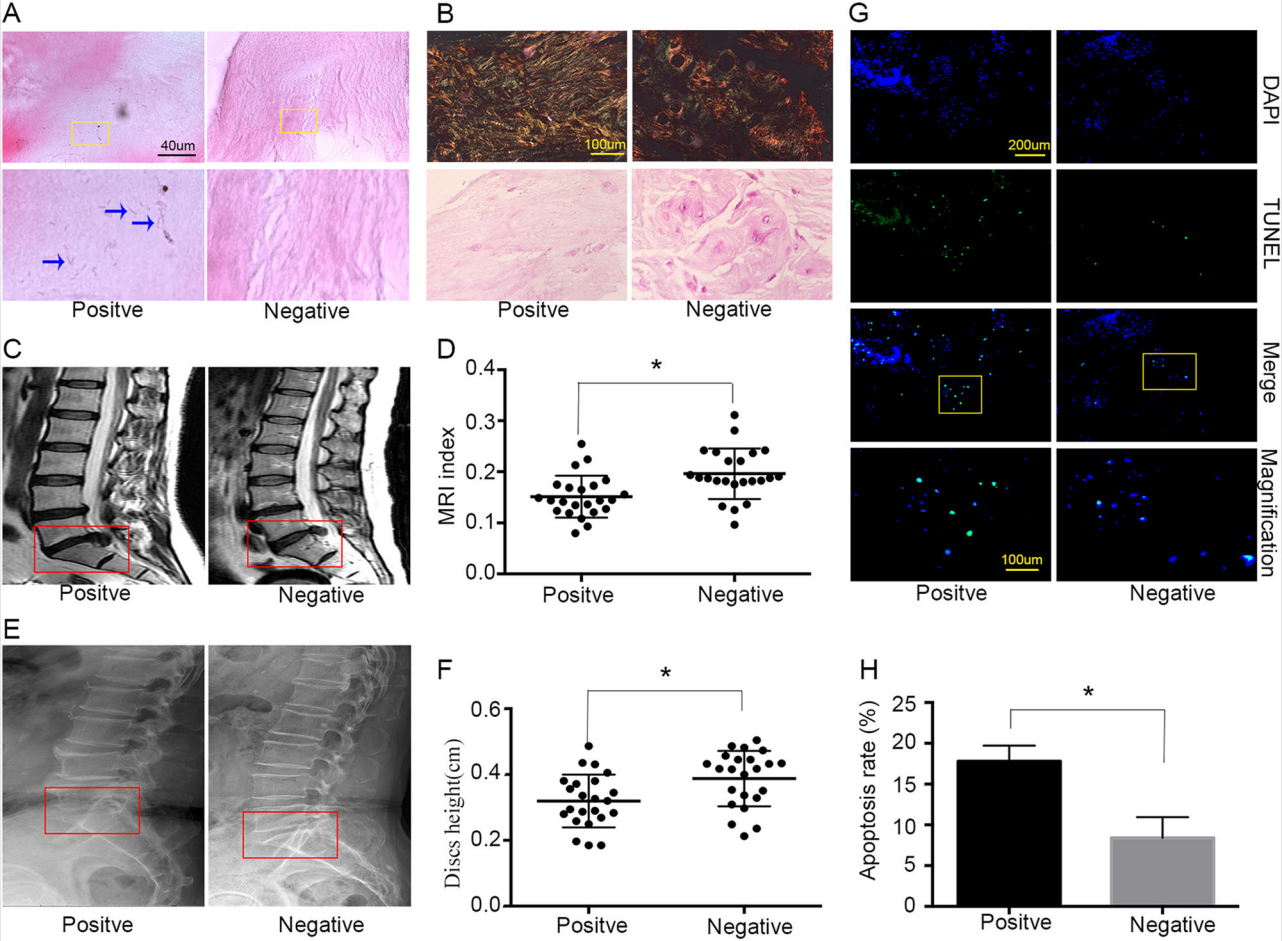
IVDs positive for *P. acnes* had more apoptotic NPCs concomitant with severe disc degeneration in patients **a** H&E staining revealed rod-shaped bacteria growing in clusters in human disc tissues (indicated by blue arrows). **b** The severity of degeneration in the annulus fibrosus and nucleus pulposus was evaluated by Picrosirius Red and Safranin-O staining, respectively. **c**–**f** Representative images from MRI (**c**) and lateral X-ray (**e**) indicated severe degeneration in *P. acnes*-positive IVDs (*n* = 23 for each group). **g**,** h** More apoptotic NPCs were found in *P. acnes*-positive IVDs by TUNEL staining (*n* = 9 for each group). *P < 0.05, *P* values were analyzed using Student’s *t* test

To investigate the mechanisms by which *P. acnes* induces IVDs, the pathological changes in *P. acnes*-infected degenerated IVDs was first examined. In total, 108 degenerated IVDs were harvested from patients, and 23 (23/106, 21.70%) were identified as *P. acnes*-positive after examination of the anaerobic culture and 16S rDNA by PCR, while 85 samples were *P. acnes*-negative. As confounding factors, the variables of age, primary symptoms, duration of symptoms, and surgery level would dramatically affect the severity of IVDD, and therefore a case-controlled matched method was used to compare the *P. acnes*-positive and *P. acnes*-negative samples according to a previous protocol, as mentioned detail in the Methods section. The morphological examination showed that the bacteria were Gram-positive, rod-shaped, and grew in a cluster (Fig. 1a).

Moreover, histological examination revealed a decrease in collagen I fibers by Picrosirius Red staining in the annulus fibrosus, as well as a reduction in glycosaminoglycan by Safranin-O staining in *P. acnes-*infected IVDs compared to *P. acnes*-negative tissues in the nucleus pulposus (Fig. 1b). A further quantitative analysis of disc degeneration severity suggested that the *P. acnes*-positive group demonstrated a significant decrease in the gray value of IVDs by MRI, observed as more hypo-intense signals in the midsagittal T2-weighted images (*P* < 0.05; Fig. 1c, d). In addition, the intervertebral heights in the *P. acnes*-positive group were also much lower than those in the *P. acnes*-negative group (*P* < 0.05; Fig. 1e, f). Taken together, these results demonstrated that *P. acnes*-infected discs had more severe IVDD than *P. acnes*-negative tissues.

Furthermore, increased numbers of apoptotic NPCs were found in *P. acnes*-positive samples compared with *P. acnes*-negative discs, as examined by TUNEL staining (*P* < 0.05, Fig. 1g, h). Hence, it was reasonable to hypothesize that *P. acnes* may deteriorate IVDD by inducing NPC apoptosis because cellular loss of NPCs is believed to play an important role in IVDD^15^.

### Infection by *P. acnes* induces disc degeneration by promoting NP cell apoptosis

**Fig. 2:**
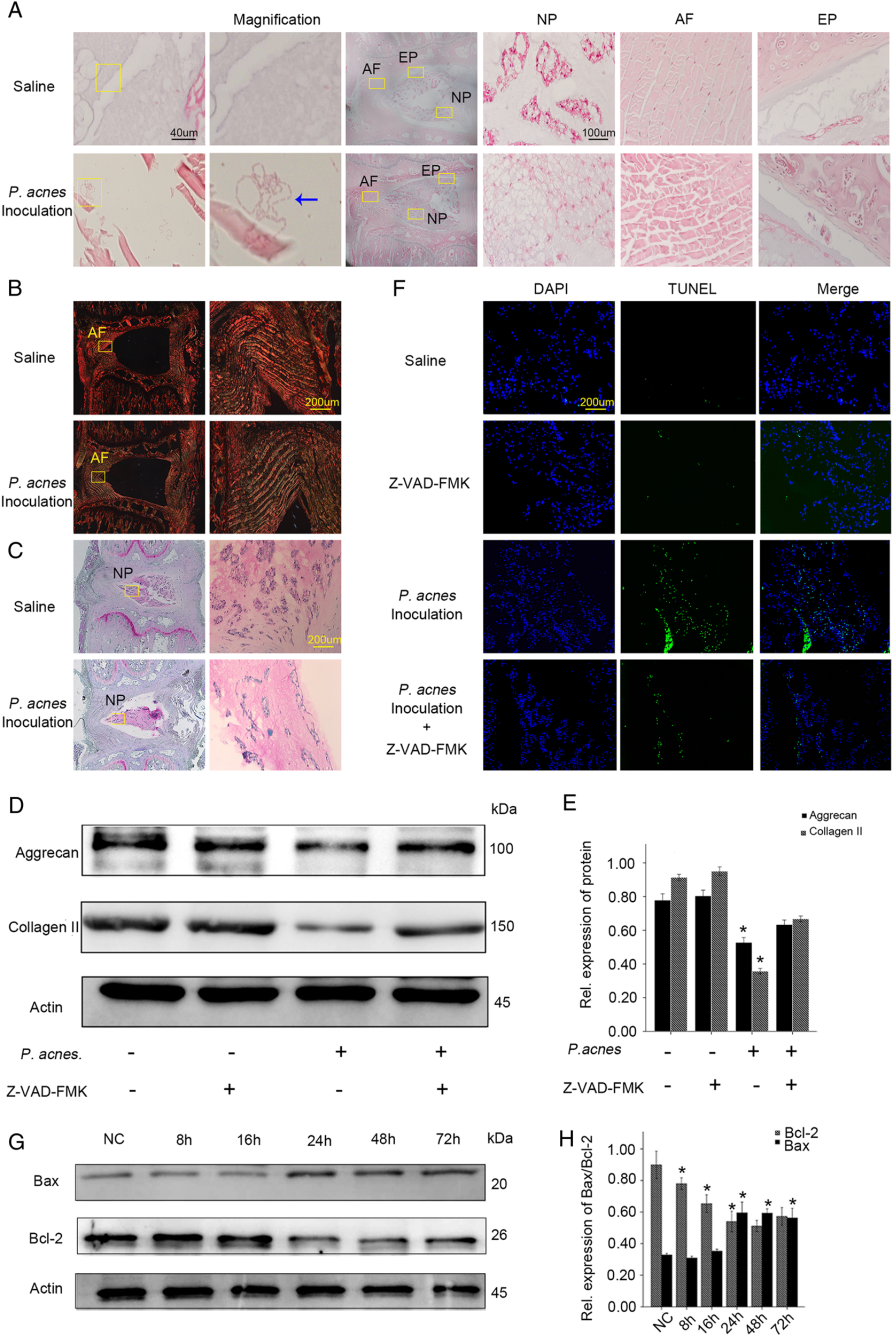
Caudal IVD inoculation with *P. acnes*-induced IVDD by promoting NPC apoptosis in rats **a** H&E staining revealed the presence of inoculated *P. acnes* inside caudal IVDs, along with serpentine and disorganized fibers in the annulus fibrosus, fewer cells and a reduced ECM in the nucleus pulposus as well as damage in endplates after inoculation for 72 h. **b**, **c** Picrosirius Red staining (**b**) and Safranin-O staining (**c**) suggested a decrease in collagen I fibers and glycosaminoglycans in *P. acne*s-inoculated IVDs. **d** Death of NPCs detected by TUNEL staining in IVDs infected with *P. acnes* treated with or without Z-VAD-FMK for 72 h. **e**, **f** Time-dependent expression of Bax and Bcl-2 induced by *P. acnes*. *The different infection groups compared with the NC group. **g**, **h** Western blot analysis of aggrecan and Collagen II in IVDs induced by *P. acnes* treated with or without Z-VAD-FMK for 72 h. *The infection groups compared with the infection+Z-VAD-FMK group. *P < 0.05, *P* values were analyzed by one-way ANOVA. Data are presented as the mean ± SD from three independent experiments (four rats per group, three discs per rat). NP nucleus pulposus, AF annulus fibrosus, EP endplate, Z-VAD-FMK caspase inhibitor

To further understand the relationship between NPC apoptosis and disc degeneration caused by *P. acnes*, the bacteria were inoculated into the caudal IVD of rats. After 72 h, rod-shaped *P. acnes* were found in the NP and AP upon histological observations (Fig. 2a). H&E staining also showed that *P. acnes*-inoculated IVDs exhibited drastic IVDD compared with saline-injected IVDs, as observed by decreased numbers of cells and ECM and disorganized cellular components in the nucleus pulposus, as well as serpentine and disordered fibers in the annulus fibrosus (Fig. 2a). In addition, the amount of collagen I fibers detected by Picrosirius Red staining and glycosaminoglycan detected by Safranin-O staining decreased abundantly in *P. acnes*-inoculated IVDs compared with saline-injected IVDs (Fig. 2b, c). Quantitative analysis suggested that the expression of aggrecan and collagen II decreased significantly in *P. acnes*-inoculated IVDs compared with the controls (Fig. 2d, e). These results demonstrated that *P. acnes*-induced severe IVDD following its colonization inside IVD.

TUNEL staining also revealed an abundant increase in NPC death in response to *P. acnes* colonization (Fig. 2f), accompanied by an increase in Bax and a decrease in Bcl-2 in a time-dependent manner (Fig. 2g, h). These results validated that the apoptosis of NPCs in vivo was caused by *P. acnes*.

More importantly, when *P. acnes*-induced apoptosis of NPCs was partly inhibited by Z-VAD-FMK (Fig. 2f), *P. acnes*-induced IVDD was partly ameliorated, as evidenced by the relative increase in expression of aggrecan and collagen II (Fig. 2d, e). Together with the evidence obtained from human tissues, we came to the rational conclusion that inoculation of *P. acnes* caused IVDD by promoting NPC apoptosis.

**Fig. 3:**
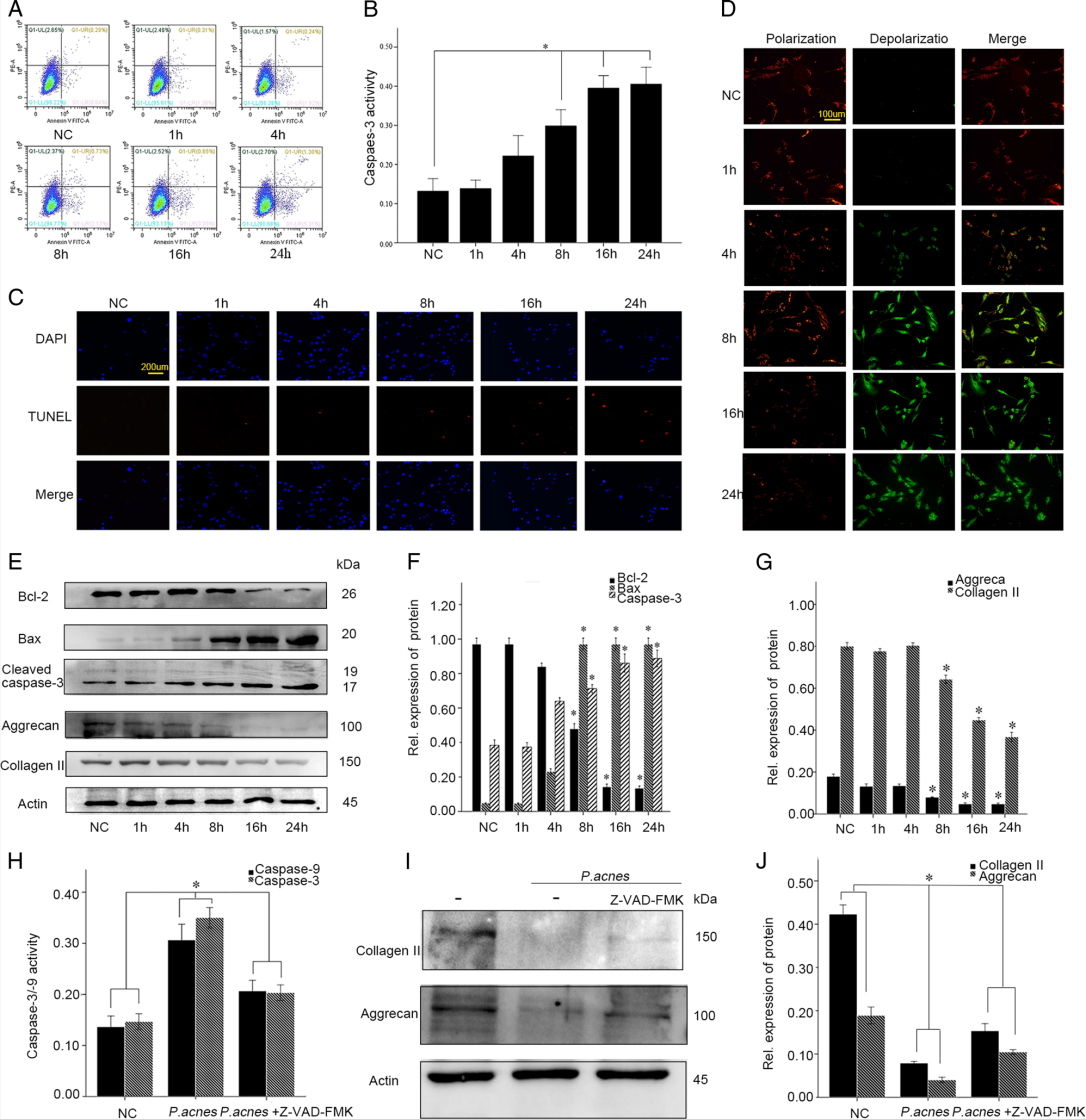
Incubation with *P. acnes* induces apoptosis of NPCs in vitro **a**–**c** Apoptotic rates of NPCs detected by flow cytometry (**a**), caspase-3 activity (**b**), and TUNEL staining (**c**) in NPCs after incubation with *P. acnes* for different periods at a MOI = 100:1. **d** Immunofluorescence analysis of JC-1 in NP cells in response to *P. acnes* infection at a MOI = 100:1. **e**–**g** Time curve expression of Bax, Bcl-2, cleaved caspase-3, aggrecan, and collagen II in NPCs induced by *P. acnes* at a MOI = 100:1. *The different infection groups compared with the NC group. **h**–**j** Western blot analysis of aggrecan and Collagen II. NPCs were treated with or without Z-VAD-FMK (20 µM) pre-incubation for 1 h and then infected with *P. acnes* for 24 h. *P < 0.05, *P* values were analyzed by one-way ANOVA. Data are presented as the mean ± SD from three independent experiments. NC negative control, Z-VAD-FMK caspase inhibitor

Next, we sought to investigate whether *P. acnes*-induced IVDD by promoting NPC apoptosis in vitro. After co-culturing the NPCs with *P. acnes* (MOI = 100) for different time periods (1, 4, 8, 16, 24 h), the apoptotic rates of the NPCs gradually increased, as detected by flow cytometry, caspase-3 activity, and TUNEL staining (Fig. 3a–c). Concomitantly, a time-dependent increase in Bax and caspase-3 expression and a decrease in Bcl-2 expression was observed (Fig. 3e, f). In parallel with the increased apoptosis, *P. acnes* infection induced significant NP cell degeneration, as demonstrated by the decrease in aggrecan and collagen II expression (Fig. 3e, g). However, these processes were partly dampened by Z-VAD-FMK (20 µM) (Fig. 3h–j). Taken together, these results revealed that *P. acnes* could induce NP cell degeneration by promoting NP cell apoptosis in vitro.

To explore the potential pathway underlying *P. acnes*-induced apoptosis of NPCs, the mitochondrial membrane potential was measured with JC-1, a specific mitochondrial dye. The results showed that the relative ratio of red fluorescence intensity/green fluorescence intensity decreased significantly in NPCs relative to the infection time of *P. acnes*. This result suggested that the abnormal changes in mitochondrial membrane potential might be a key contributor to *P. acnes*-induced apoptosis (Fig. 3d). In addition, the altered expression of Bcl-2 and Bax also supported the mitochondrial-mediated apoptosis pathway (Fig. 3e, f). These results revealed that the mitochondrial-mediated apoptosis was key for the *P. acnes*-induced apoptosis of NPCs.

### Apoptosis of NPCs by *P. acnes* was caused via the TLR2/JNK/ mitochondrial-mediated pathway

**Fig. 4:**
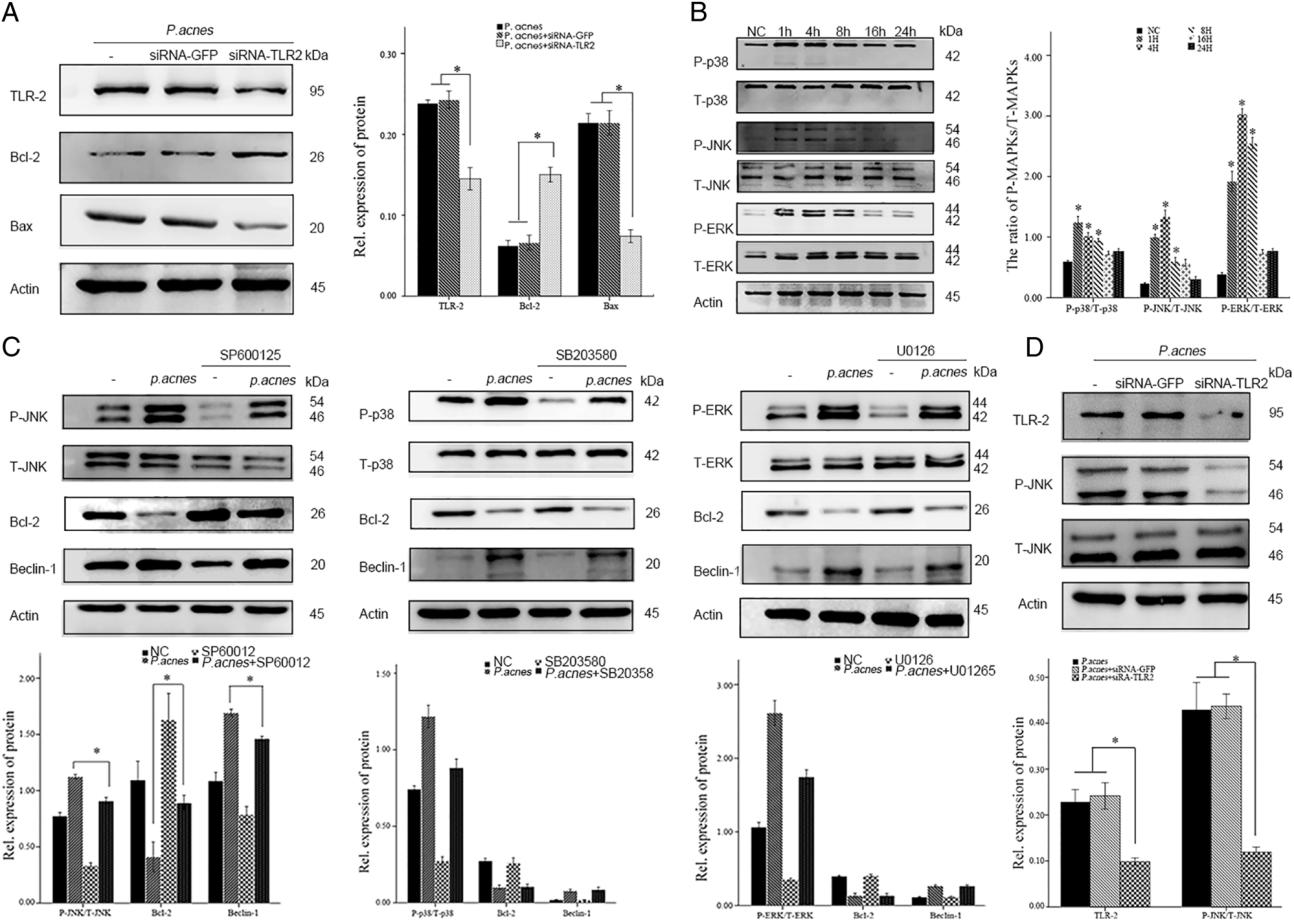
Infection with *P. acnes* induces NPC apoptosis via the TLR2/JNK pathway **a** Western blot analysis of TLR2, Bcl-2 and Bax. NPCs were per-transfected with or without *Tlr2*-siRNA for 16 h and then infected with *P. acnes* for 8 h. **b** Western blot analysis of MAPKs (ERK, JNK, p-38) in NPCs induced by *P. acnes* for different time periods. *The different infection groups compared with the NC group. **c** Western blot analysis of MAPKs (ERK, JNK, p-38) and Bcl-2/beclin-1. NPCs were per-incubated for 2 h with or without MAPKs specific inhibitors (SP600125 20 µM, SB203580 10 µM, U0126 20 µM) in the presence or absence of *P. acnes* for 1 h (for ERK, JNK, p-38) and 24 h (for Bcl-2/Beclin-1). **d** Western blot analysis of TLR2, P-JNK, and T-JNK. NPCs were per-transfected with or without *Tlr2*-siRNA for 16 h and then infected with *P. acnes* for 1 h. *P < 0.05, *P* values were analyzed by one-way ANOVA. Data are presented as the mean ± SD from three independent experiments

The molecular mechanism involved in *P. acnes*-induced apoptosis of NPCs was then explored. Since Toll-like receptor 2 (TLR2) is a well-known receptor for gram-positive bacteria, we analyzed whether TLR2 activation was required for *P. acnes*-induced NPC apoptosis. The results revealed that silencing TLR2 expression using a specific siRNA and competing for TLR2 binding using a TLR2 antagonist CU-CPT22^20^ (10 µM, Sigma-Aldrich, Germany) significantly dampened the *P. acnes*-induced increase in Bax and cleaved caspase-3, as well as the decrease in Bcl-2, suggesting that TLR2 played an important role in *P. acnes*-induced apoptosis of NPCs (Fig. 4a and Supplementary Figure S1A).

Next, TLR2-mediated downstream signaling pathways, including p38MAPK, JNK, ERK, and NF-κΒ were investigated^21^. Phosphorylation of P38, JNK, ERK, and NF-κΒ increased dramatically after *P. acnes* infection for 1 h, peaking at 4 h, and then gradually declined at 8, 16, and 24 h (Fig. 4b and Supplementary Figure S1B). However, following inhibition with the corresponding inhibitors, only one was found to be effective; SP600125 (20 µM, NO S1876, Beyotime, Shanghai, China), the JNK pathway inhibitor, significantly restored the *P. acnes*-induced decrease in Bcl-2 and increase in Beclin-1. However, SB203580 (10 µM, NO S1863, Beyotime, Shanghai, China), the P38 inhibitor, U0126 (20 µM, NO S1901, Beyotime, Shanghai, China), the ERK inhibitor, and BAY11-7082 (10 µM, NO S1523, Beyotime, Shanghai, China), the NF-κΒ inhibitor, had no effect on this process (Fig. 4c and Supplementary Figure S1C). Furthermore, the phosphorylation of JNK induced by *P. acnes* was blocked by TLR2-siRNA (Fig. 4d). Taken together, these data demonstrated that *P. acnes*-induced apoptosis of NPCs occurs via the TLR2–JNK pathway.

### **Autophagy promoted*P. acnes*-induced apoptosis**

**Fig. 5:**
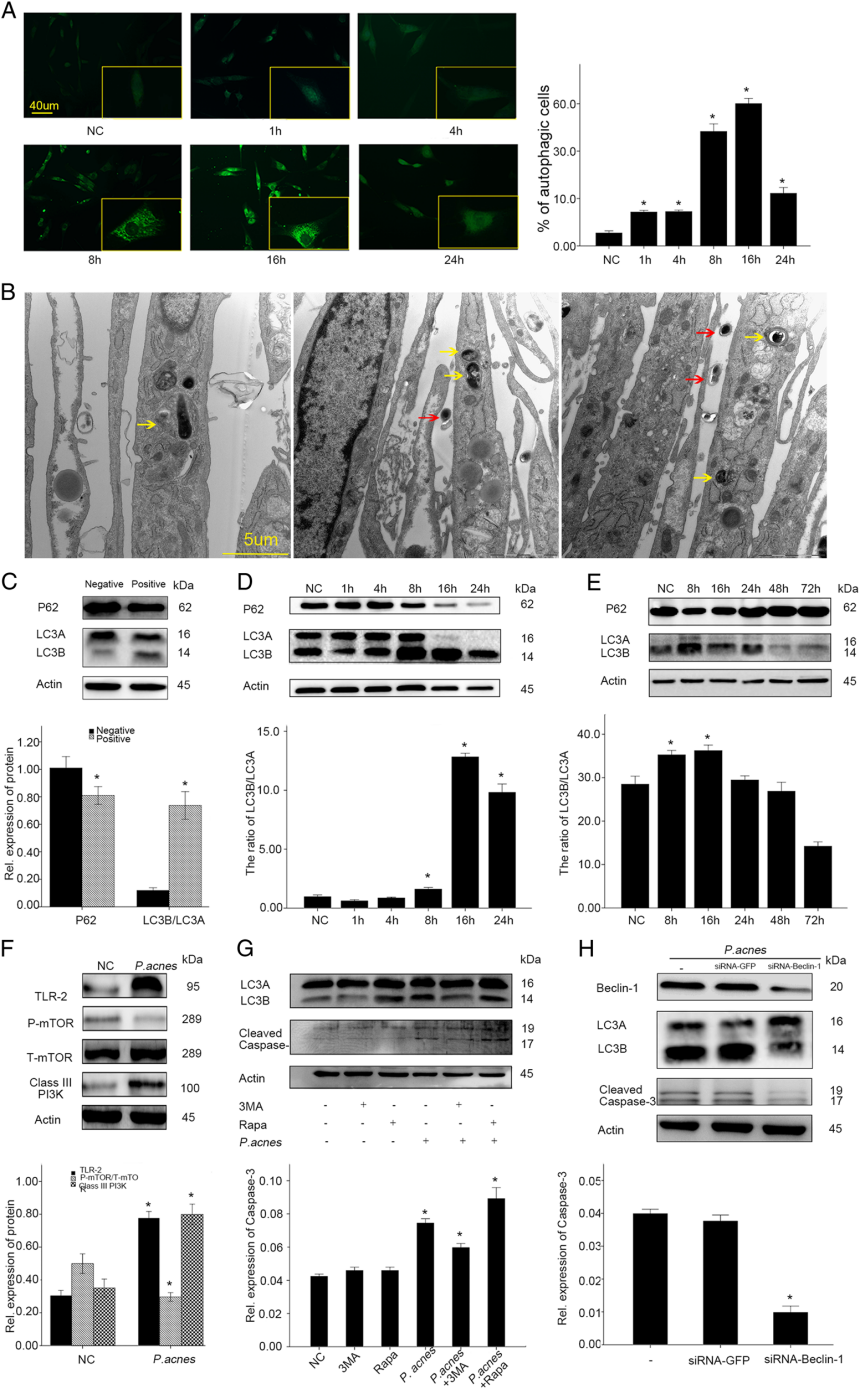
Autophagy promotes *P. acnes*-induced apoptosis **a** Immunofluorescence analysis of LC3 in NPCs induced by *P. acnes* at a MOI = 100:1 for different time periods. *The different infection group compared with the NC group. **b** Electron microscopy images of autophagy. NPCs infected with *P. acnes* at a MOI 100 were examined at 8 h post-infection. Intracellular *P. acnes* were surrounded by a variety of membrane structures (indicated by a yellow arrow), and extracellular *P. acnes* are indicated by a red arrow. **c** Western blots of p62 and LC3 in *P. acnes-*positive and negative nucleus pulposus (*n* = 9 for each group). *Positive sample vs. negative sample. **d** Western blots of p62 and LC3 in NPCs induced by *P. acnes* at s MOI = 100:1 for different time periods. *The different infection groups vs. the NC group. **e** Western blots of p62 and LC3 in rat IVDs after infection with *P. acnes* for different time periods. (*n* = 4 rats per group, three discs per rat). * The different infection groups vs. the NC group. **f** Western blot analysis of the phosphorylation of mTOR and the expression of TLR2/Class III PI3K in NPCs after 1 h of *P. acnes* infection (MOI = 100:1). *P. acnes group vs. negative control group. **g** Western blot analysis of LC3 and cleaved caspase-3 in NPCs induced by *P. acnes* at a MOI = 100:1 for 8 h pretreated with 3-MA (5 mM) or rapamycin (500 μM) for 2 h. *P. acnes group vs. the *P. acnes* + 3MA/Rapa group. **h** Western blot analysis of Beclin-1, LC3, and cleaved caspase-3. NPCs were pre-transfected with or without *Beclin-1*-siRNA for 16 h = and then infected with *P. acnes* for 8 h. *The *P. acnes* + siRNA-*Beclin-1* group vs. the *P. acnes* or *P. acnes* + siRNA-GFP group. *P < 0.05, *P* values were analyzed by one-way ANOVA. Data are presented as the mean ± SD from three independent experiments

Autophagy has been shown to be a new mechanism that causes apoptosis of NPCs^22^. To investigate whether autophagy was also involved in the *P. acnes*-induced apoptosis, the autophagy of NPCs was examined after co-culture with *P. acnes*. Immunofluorescence analysis showed that autophagosomes and autolysosomes were rarely detected in the controls, while their levels increased gradually after infection with *P. acnes*, as demonstrated by an increase in the punctate fluorescence staining signals observed for LC3 in the cytoplasm of NPCs after 8 h of incubation, which peaked at 16 h (Fig. 5a). Autophagy of foreign entities, such as bacteria, viruses, and other pathogens, is termed xenophagy^23^. To determine the occurrence of xenophagy, it is essential to validate the presence of *P. acnes* in the membrane^24^. Electron microscope examination of NPCs at 8 h post-infection (MOI = 100:1) revealed a variety of membrane structures containing *P. acnes* (Fig. 5b). Furthermore, conversion of LC3A to LC3B is essential for autophagosome formation, and the western blot results showed that *P. acnes* infection increased the ratio of LC3B/LC3A, but it decreased the levels of p62 in *P. acnes*-positive tissues (Fig. 5c). p62 is another autophagy marker that functions as the LC3B-binding protein that bundles ubiquitinated proteins that are aggregated in the autophagosome. Interestingly, a similar trend was found for p62 in *P. acnes*-infected NPCs and *P. acnes*-inoculated IVD; moreover, *P. acnes*-activated autophagy in vitro and in vivo were further validated by the conversion of LC3A to LC3B (Fig. 5d, e, Supplementary Figure S2A and S2B). In addition, we detected a decrease in mTOR phosphorylation as well as an increase in class III PI3K in NPCs after 1 h of *P. acnes* infection (Fig. 5f). Next, we blocked mTOR and class III PI3K with rapamycin (500 µM, NO V900930, Sigma, Germany) and 3-MA (5 nM, NO M9281, Sigma, Germany), respectively. The results showed that rapamycin pretreatment heightened, whereas 3-MA pretreatment dampened, *P. acnes*-induced autophagy activation (Fig. 5g and Supplementary Figure S2C). These data revealed that *P. acnes* could induce autophagy activation in NPCs through the PI3K and mTOR pathway.

A possible relationship between autophagy and apoptosis was subsequently investigated. The loss of function experiment revealed that the autophagy inhibitor, 3MA, decreased the conversion of LC3A to LC3B, as well as the cleavage of caspase-3. In contrast, the gain of function experiment showed that activation of autophagy using rapamycin significantly promoted *P. acnes*-induced caspase-3 cleavage (Fig. 5g and Supplementary Figure S2C). In addition, as Beclin-1, a core component of the autophagy machinery, plays a central role in the regulation of autophagy^25^, we knocked down *Beclin-1* via siRNA. The results showed that, consistent with the decrease in Beclin-1 expression, the conversion of LC3A to LC3B and the cleavage of caspase-3 were also dampened by *Beclin-1* siRNA transfection (Fig. 5h and Supplementary Figure S2D–S2E). Taken together, these results demonstrated that autophagy could play an auxiliary role in *P. acnes*-induced apoptosis of NPCs.

## Discussion

In the present study, *P. acnes* was shown to colonize non-pyogenic IVD based on bacterial culture and histological examination, with a prevalence of 21.70%, similar to previous studies that reporting a prevalence ranging from 13 to 44%^4,26^. Some researchers have argued that *P. acnes* isolated from IVD may be contaminants stemming from the skin during tissue harvest or culture^27^. However, we have shown herein that most of the isolated *P. acnes* were likely to represent original growth from the discs due to the following reasons. First, more than ten research groups have independently demonstrated the existence of *P. acnes* in bacterial culture^7^ by molecular analysis^28^ and histological examination^3,13^. One study was robust, with a sample size as large as 368 patients and a positive rate of 32.33% for *P. acnes* infection^3^. Thus, it is not reasonable to attribute all isolated *P. acnes* as contaminants. Second, two recent histological reports verified that *P. acnes* grew in clusters within the tissues rather than on the surface of tissues as dispersed single cells, indicating that the bacterium grew inside IVD for a long time^3,13^. Finally, the negative culture results from surrounding muscle and ligaments indicated that most of the IVDs were harvested under sterile conditions, and the possibility of contamination was low^9^. In summary, the latent existence of *P. acnes* inside non-pyogenic IVD should not be ignored or overlooked, and its pathological role in IVD requires a comprehensive analysis.

The main pathological role of latent colonized *P. acnes* was the induction of IVDD. In previous studies, inoculation of *P. acnes* initiated or accelerated IVDD in animals^8,11^. Moreover, epidemiological studies found a possible relationship between latent infection of *P. acnes* and IVDD^9^. In the present study, *P. acnes*-positive human samples had severe IVDD, both quantitatively and through histological analysis. Additionally, inoculation of *P. acnes* into the caudal IVD of rats further confirmed the bacteria-induced IVDD. Thus, there is sufficient evidence supporting the ability of *P. acnes* to induce IVDD.

Furthermore, *P. acnes* was found to cause apoptosis of NPCs both in vivo and in vitro. Previous studies have suggested that *P. acnes* have the ability to induce apoptosis in other cells. For example, apoptosis of THP-1 monocytic cells increased when co-cultured with *P. acnes*^29^. In addition to direct effects, *P. acnes* was shown to secrete lipopolysaccharides, which are toxic factors that induce apoptosis of hepatocytes^30^. Here, the results of TUNEL staining showed that apoptotic rates were significantly higher in *P. acnes-*positive than negative samples from patients. Similarly, in animal experiments, *P. acnes* inoculation induced apoptosis of NPCs. In in vitro experiments, apoptosis of NPCs induced by *P. acnes* was obvious and time-dependent. Thus, the apoptotic ability of *P. acnes* in NPCs was confirmed.

Typically, apoptosis of NPCs is considered to be a host mechanism leading to IVDD^14,15,31^. NPCs form the core cell population that maintains the integrity and bio-activity of IVD because they synthesize and secrete the ECM, which protects against IVDD caused by mechanical stress^32^. In contrast, the onset of NPC apoptosis predicts the beginning of IVDD^16^. Thus, *P. acnes*-induced apoptosis of NPCs could be considered a main mechanism responsible for IVDD. Clinical data suggested that *P. acnes*-infected human IVD had more apoptotic NPCs concomitant with severe disc degeneration in patients. Additionally, inoculation of *P.* *acnes*-induced apoptosis of NPCs simultaneously with disc degeneration in animal models. More importantly, pretreatment with the inhibitor of apoptosis significantly ameliorated *P. acnes*-induced IVDD. Therefore, a rational conclusion could be drawn that *P. acnes* induces IVDD by promoting apoptosis of NPCs.

In this study, TLR2 was the cellular receptor of NPCs that responded to *P. acnes* infection. As one of the pathogen recognition receptors, TLR2 recognizes microorganisms, especially Gram-positive bacteria^33,34^. A previous study suggested that TLR2 is the primary receptor in monocytes that responds to stimulation by *P. acnes*^35^. This receptor was also expressed at the cellular surface of NPCs and was shown to play crucial roles in the response of NPCs to various stimuli^36^. Thus, it was not surprising that TLR2 participated in the signaling pathway when *P. acnes* interacted with NPCs.

Moreover, TLR2 is known to regulate several key downstream proteins, such as JNK, in cells^37,38^. JNK belongs to a widely conserved family of serine/threonine protein kinases that are implicated in many cellular processes, such as proliferation, differentiation and apoptosis^39^. After activation of TLR2, JNK is activated by MyD88^40^ and subsequently triggers apoptosis via the mitochondrial or nuclear pathway^41^.

The mitochondria-mediated apoptotic pathway is one of the major pathways involved in JNK-regulated cellular apoptosis, and NPC apoptosis also relates to this pathway^17,42^. The pathway requires the inhibition of Bcl-like proteins (Bcl-2, Bcl-XL), which mainly reside in mitochondria, and conformational changes in Bax that induce permeability of the mitochondrial membrane^41,43,44^. The mitochondria then commit cells to apoptosis by releasing cytochrome c, Smac/Diablo, AIF, and activating procaspase-9/-3^45^. Caspases are synthesized as inactive precursors that must be cleaved autocatalytically or by other caspases to be activated. In this study, the mitochondrial membrane potential was down-regulated after incubation with *P. acnes* in a time-dependent manner, and the expression of Bcl-2 and Bax significantly decreased or increased. These results demonstrated that the mitochondrial pathway could be involved in *P. acnes*-induced apoptosis of NPCs.

Moreover, we found that *P. acnes* was capable of activating autophagy in NPCs. Autophagy is a conserved function in many cells, and NPCs also use this function to counteract different types of harmful extracellular stimuli^22^. A previous study has suggested that *P. acnes* have the ability to induce autophagy in cell lines of macrophages (Raw264.7), mesenchymal cells (MEF), and epithelial cells (HeLa)^46^. However, *P. acnes*-induced autophagy in NPCs was not demonstrated. In the present study, immunofluorescence and protein expression of LC-3B and p62 verified this process in NPCs. Interestingly, electron microscopy observations suggested that the autophagy induced by *P. acnes* occurred via a selective type of autophagy, xenophagy, which is an evolutionarily conserved mechanism that is classically observed after host cell invasion^47^.

The effects of autophagy in NPCs are complicated and diverse during the maintenance of homeostasis in IVD. Some studies have suggested that this phenomenon occurs as a protective mechanism^48^, while others have insisted that the process deteriorates IVDD through many pathways, for example, by promoting apoptosis^49^. Among the mechanisms, autophagy and apoptosis share the same set of regulatory proteins, with Bcl-2 playing a key dual role in the control of apoptosis and autophagy^50^. Here, the loss of function and gain of function experiments suggested that apoptosis of NPCs increased or decreased significantly when treated with an inhibitor or activator of autophagy. Therefore, *P. acnes*-induced autophagy was believed to synergistically regulate *P. acnes*-induced apoptosis in NPCs.

In addition, we detected NF-κB pathway activation in response to *P. acnes*. The western blot results revealed that incubation with *P. acnes*-induced p65 phosphorylation in a time-dependent manner (Supplementary Figure S1B). Next, we blocked p65 pathway activation with the p65 pathway inhibitor BAY11 (Supplementary Figure S1C). In contrast to the dampened *P. acnes* increase in p65 phosphorylation, BAY11 pretreatment partially, but significantly, restored the decrease in type II collagen and aggrecan induced by *P. acnes*. Simultaneously, the results also showed that BAY11 pretreatment had no effect on *P. acnes*-mediated Bcl-2 and Beclin-1 expression, which suggested that NF-κB signaling pathway was involved in *P. acnes*-induced IVDD rather than in *P. acnes*-induced apoptosis and autophagy of NPCs. Interestingly, we found that incubation with *P.* *acnes*-induced IL-1β and TNF-α expression in a time-dependent manner (Supplementary Figure S1D), and BAY11 pretreatment abundantly inhibited *P. acnes*-induced IL-1β and TNF-α expression (Supplementary Figure S1E). Since the increase in IL-1β and TNF-α have been shown to induce IVDD^51^, we speculated that NF-κB signaling pathway mediated *P. acnes*-induced IVDD by regulating IL-1β and TNF-α expression.

Furthermore, in addition to the mitochondrial-mediated pathway, we found that *P. acnes* could induce NPC apoptosis through the cell death receptor-mediated extrinsic pathway. The expression of Fas, FasL and caspase-8 in *P. acnes*-positive nucleus pulposus tissues was increased in comparison to negative tissues (Supplementary Figure S3A). Similarly, incubation with *P. acnes* increased the mRNA and protein expression of Fas, FasL and Caspase-8 in NPCs in a time-dependent manner (Supplementary Figure S3B and S3C).

The limitations of this study are also noted. First, the TLR2/JNK/mitochondrial-mediated apoptotic pathway was verified only in vitro, but not in vivo. Similarly, autophagy-regulated apoptosis after *P. acnes* stimulation was shown only in vitro and more evidence for this process should be provided in vivo in future studies. In addition, the component of the *P. acnes* that activated TLR2 to induce apoptosis of NPCs was not demonstrated in this study.

**Fig. 6:**
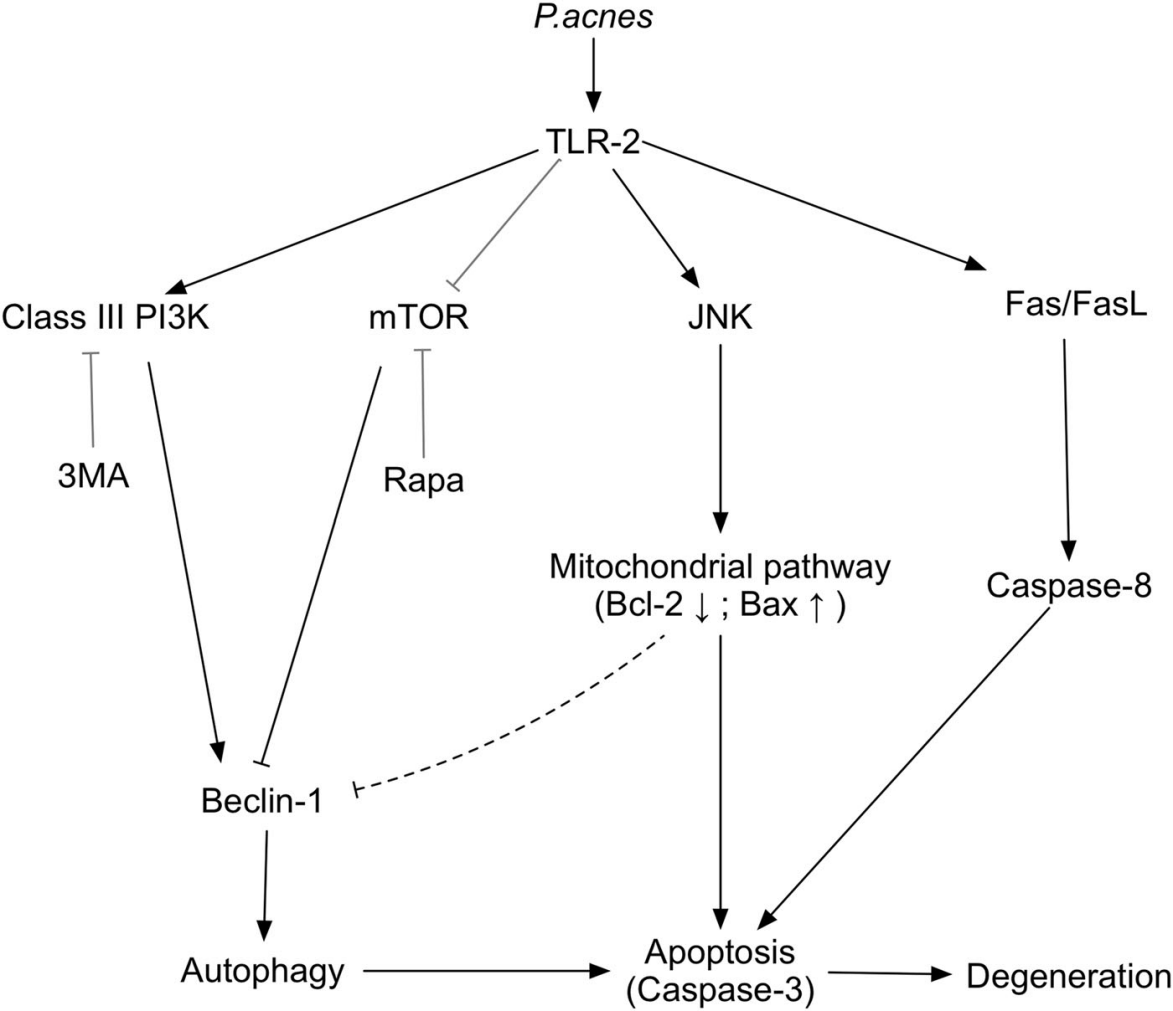
Schematic illustration of *P. acnes*-induced IVDD by promoting apoptosis and autophagy

In conclusion, this study demonstrated that *P.* *acnes*-induced IVDD by promoting apoptosis of NPCs via the TLR2/JNK/mitochondrial-mediated apoptotic pathway and autophagy (Fig. 6). The confirmation of *P. acnes* as a pathogenic factor for IVDD and elucidation of the underlying mechanisms provide new insights into IVDD and may ultimately lead to the development of novel treatment regimens for spinal disease.

## Electronic supplementary material

Supplementary Figure S1

Supplementary Table S1

Supplementary figure legends

Supplementary Figure S2

Supplementary Figure S3
