# Supplementary figures and images for: Propionibacterium acnes induces intervertebral disc degeneration by promoting nucleus pulposus cell apoptosis via the TLR2/JNK/mitochondrial-mediated pathway
Source: Emerg Microbes Infect. 2018 Jan 10;7:1. doi: 10.1038/s41426-017-0002-0 (PMC5837142; doi:10.1038/s41426-017-0002-0)

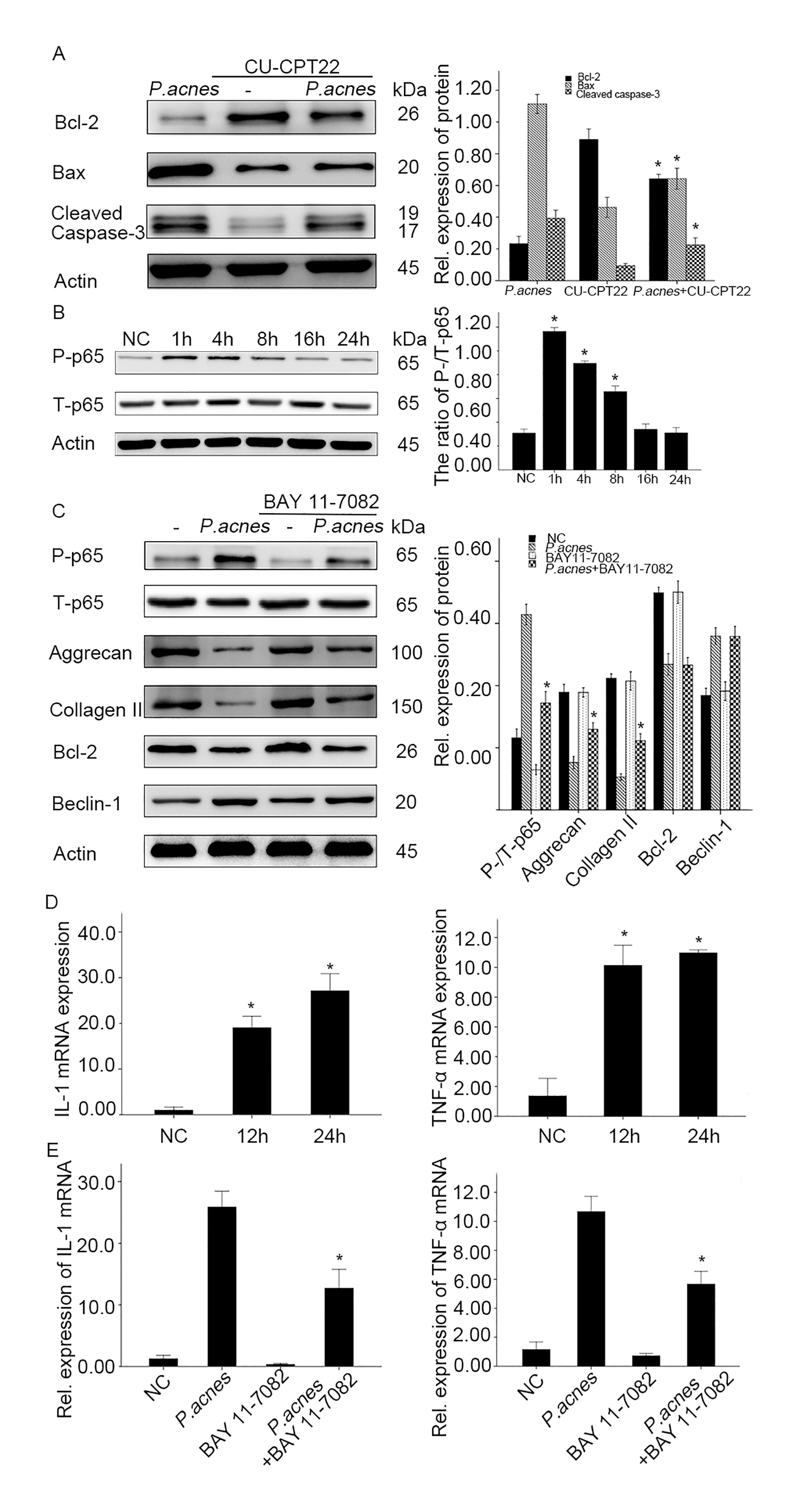

Supplement: Supplementary file 1 — Supplementary Figure S1 [file 41426_2017_2_MOESM1_ESM.tif]

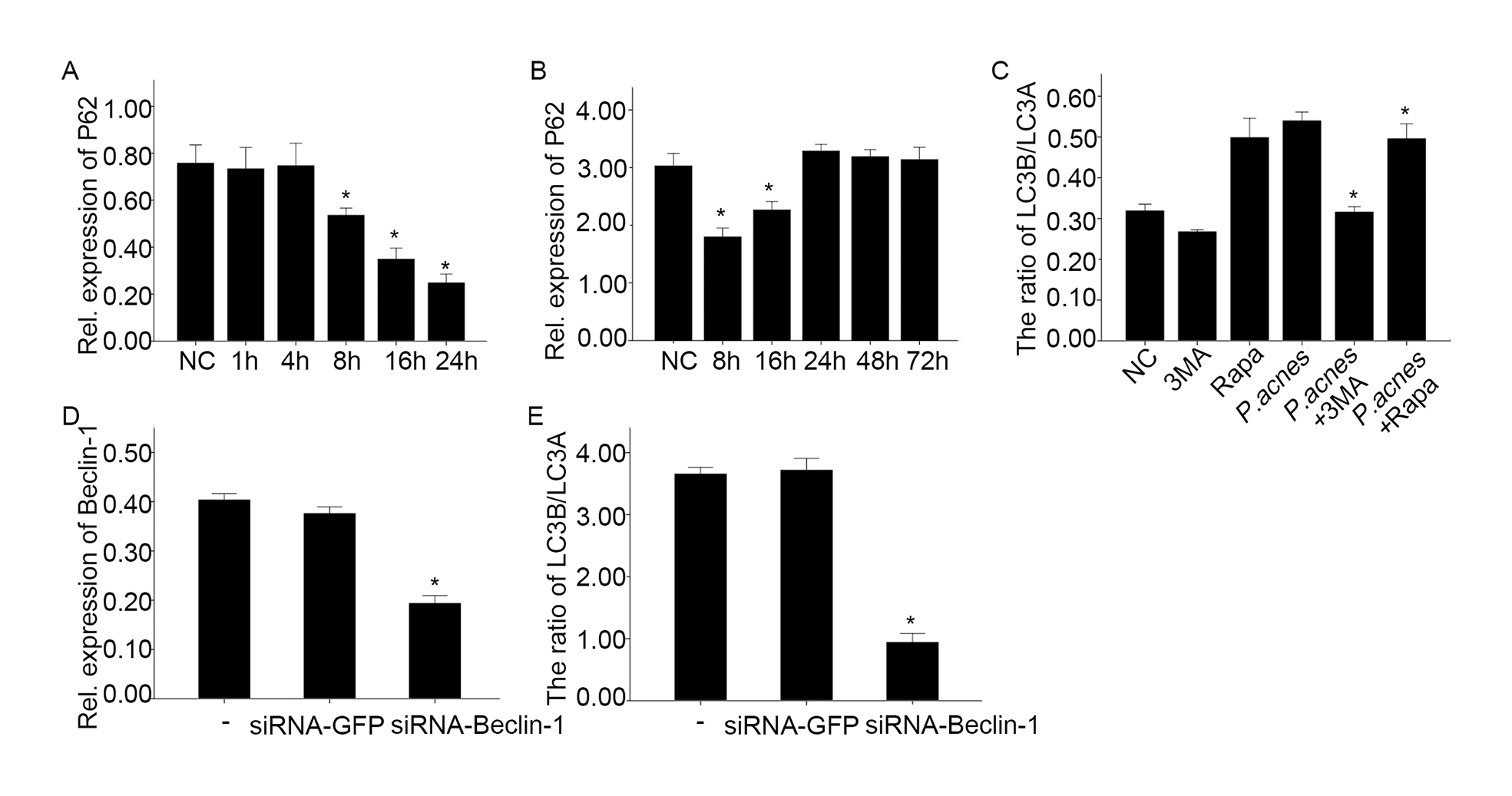

Supplement: Supplementary file 4 — Supplementary Figure S2 [file 41426_2017_2_MOESM4_ESM.tif]

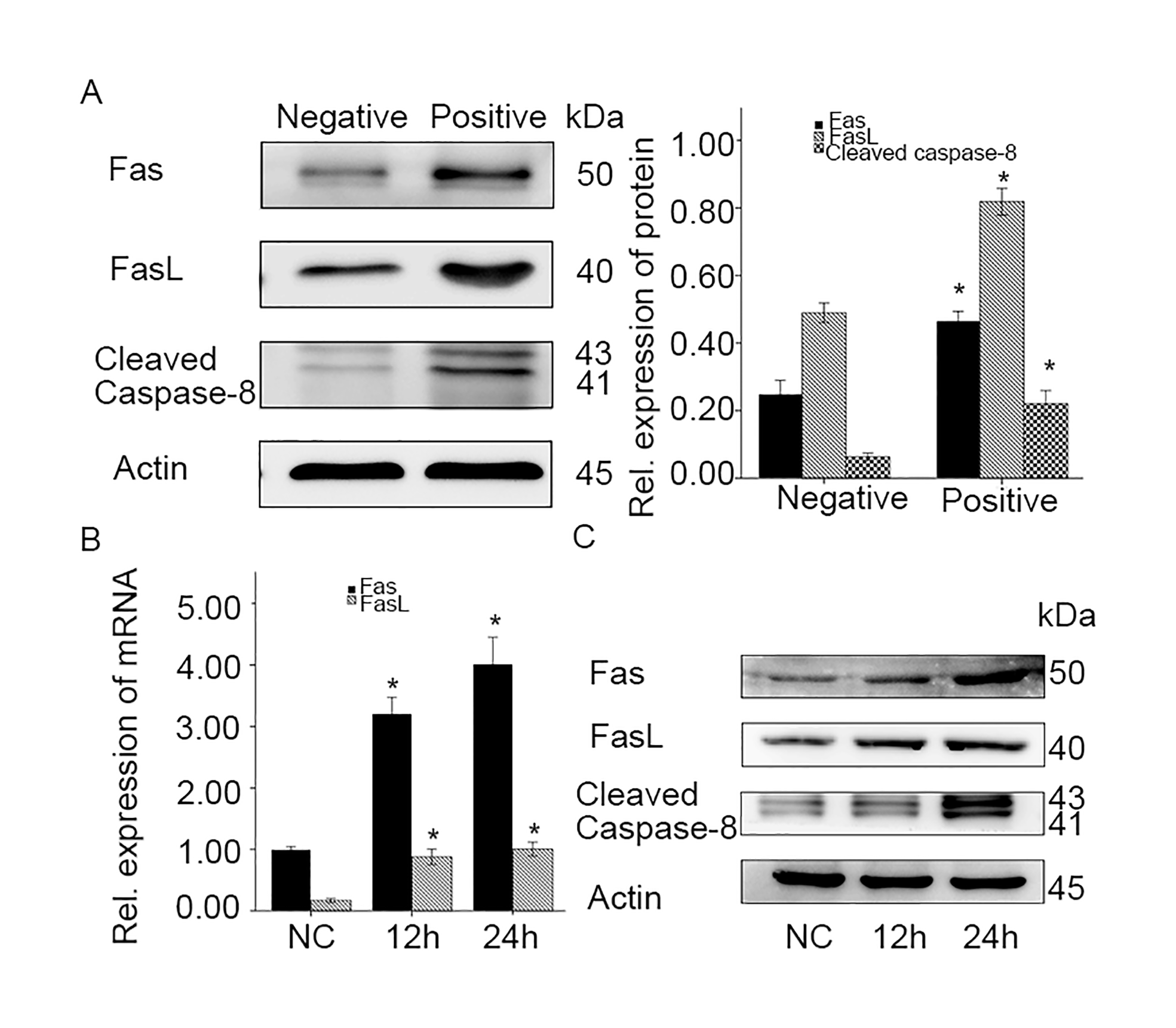

Supplement: Supplementary file 5 — Supplementary Figure S3 [file 41426_2017_2_MOESM5_ESM.tif]
