# Supplementary material for: Propionibacterium acnes induces intervertebral disc degeneration by promoting nucleus pulposus cell apoptosis via the TLR2/JNK/mitochondrial-mediated pathway
Source: Emerg Microbes Infect. 2018 Jan 10;7:1. doi: 10.1038/s41426-017-0002-0 (PMC5837142; doi:10.1038/s41426-017-0002-0)
Supplement: Supplementary file 2 — Supplementary Table S1 [file 41426_2017_2_MOESM2_ESM.docx]

|  | ***P. acnes* -Positive Group** | **Matched *P. acnes* -Negative Group** | ***P*-value** |
| --- | --- | --- | --- |
| Numbers of patients | 23 | 23 |  |
| Gender |  |  |  |
| Males | 10 | 10 |  |
| Females | 13 | 13 |  |
| Ages (years) | 52.73±16.03 | 52.21±15.52 | *P*<0.01 |
| Duration of symptoms (months) | 10.09±6.55 | 10.00±6.60 | *P*<0.01 |
| Levels of surgery |  |  |  |
| L3-4 | 2 | 2 |  |
| L4-5 | 10 | 10 |  |
| L5-S1 | 11 | 11 |  |

**Supplementary Table S1 Demographic information of included patients**
