# Supplementary material for: Propionibacterium acnes induces intervertebral disc degeneration by promoting nucleus pulposus cell apoptosis via the TLR2/JNK/mitochondrial-mediated pathway
Source: Emerg Microbes Infect. 2018 Jan 10;7:1. doi: 10.1038/s41426-017-0002-0 (PMC5837142; doi:10.1038/s41426-017-0002-0)
Supplement: Supplementary file 3 — Supplementary figure legends [file 41426_2017_2_MOESM3_ESM.docx]

**Supplementary Figure S1 Infection with *P. acnes* induced NPCs degeneration and inflammation via the NF-κΒ pathway.** (**A**) Western blotting analysis of Bcl-2, Bax and cleaved caspase-3. NPCs were pre-incubated for 1h with 10 μM CU-CPT22 and then treated with *P. acnes* (MOI 100) for 24h. (**B**) Western blot analysis of NF-κΒ in NPCs induced by *P. acnes* for different time periods. *The different infection groups *vs.* the NC group. (**C**) Western blot analysis of NF-κΒ, type II collagen, aggrecan, and Bcl-2/Beclin-1. NPCs were per-incubated for 2 hours with or without BAY11 in the presence or absence of *P. acnes* for 1 hour (NF-κΒ) and 24 hours (type II collagen, aggrecan, and Bcl-2/beclin-1). * *P. acnes* group *vs*. *P. acnes*+BAY 11 group. (**D**) RT-qPCR analysis of IL-1 β and TNF-α in NPCs induced by *P. acnes* for different time periods. *The different infection groups *vs*. the NC group. (**E**) RT-qPCR analysis of IL-1 β and TNF-α. NPCs were per-incubated for 2 hours with or without BAY11 in the presence or absence of *P. acnes* for 24 hours. **P. acnes* group *vs.* *P. acnes*+BAY 11 group. **P<*0.05, *P* values were analyzed by one-way ANOVA. Data were presented as mean ± SD from three independent experiments.

**Supplementary Figure S2 *P. acnes* induces NPCs apoptosis via the cell death receptor-mediated extrinsic pathway.** (**A**) Western blots of Fas, FasL and cleaved caspase-8 in *P. acnes* positive and negative nucleus pulposus. (9 for each group) (**B** and **C**) PCR and Western blot analysis of Fas, FasL and cleaved caspase-8 in NPCs induced by *P. acnes* for different time periods. **P<*0.05, the different infection groups *vs.* the NC group. *P* values were analyzed by one-way ANOVA. Data were presented as mean ± SD from three independent experiments.

**Supplementary Figure S3 The quantitative analysis of autophagy-associated protein.** **P<*0.05, *P* values were analyzed by one-way ANOVA. Data were presented as mean ± SD from three independent experiments.
